# Association between the triglyceride glucose (TyG) index and the risk of acute kidney injury in critically ill patients with heart failure: analysis of the MIMIC-IV database

**DOI:** 10.1186/s12933-023-01971-9

**Published:** 2023-08-31

**Authors:** Zewen Yang, Hongxia Gong, Fuqiang Kan, Ningning Ji

**Affiliations:** Department of Cardiology, Yiwu Central Hospital, 699 Jiangdong Road, Yiwu, 322000 Zhejiang China

**Keywords:** Triglyceride-glucose index, Insulin resistance, Heart failure, Acute kidney injury, MIMIC-IV database

## Abstract

**Background:**

Insulin resistance (IR) can be effectively assessed using the dependable surrogate biomarker triglyceride-glucose (TyG) index. In various critical care contexts, like contrast-induced acute kidney injury (AKI), an elevated TyG index has demonstrated a robust correlation with the incidence of AKI. Nonetheless, the potential of the TyG index to predict AKI in critically ill patients with heart failure (HF) remains uncertain.

**Methods:**

A cohort of participants was non-consecutively selected from the Medical Information Mart for Intensive Care IV (MIMIC-IV) database and divided into quartiles based on their TyG index values. The incidence of AKI was the primary outcome. The secondary endpoint was in-hospital mortality within both the whole study population and the subset of AKI patients. The use of the renal replacement therapy (RRT) which represented the progression of AKI severity was also included as a secondary endpoint representing renal outcome. A restricted cubic splines model and Cox proportional hazards models were utilized to evaluate the association of TyG index with the risk of AKI in patients with HF in a critical condition. Kaplan-Meier survival analysis was employed to estimate primary and secondary endpoint disparities across groups differentiated by their TyG index.

**Results:**

This study included a total of 1,393 patients, with 59% being male. The incidence of AKI was 82.8%. Cox proportional hazards analyses revealed a significant association between TyG index and the incidence of AKI in critically ill patients with HF. The restricted cubic splines model illustrated the linear relationship between higher TyG index and increased risk of AKI in this specific patient population. Furthermore, the Kaplan-Meier survival analyses unveiled statistically significant differences in the use of RRT across the subset of AKI patients based on the quartiles of the TyG index.

**Conclusions:**

The results highlight the TyG index as a robust and independent predictor of the incidence of AKI and poor renal outcome in patients with HF in a critical condition. However, further confirmation of causality necessitates larger prospective studies.

## Introduction

Despite the approval of many new drugs for heart failure (HF) over the last years, the rates of morbidity and mortality among patients with HF remain high [1]. Acute kidney injury (AKI) is a common complication in these patients, particularly those who require admission to the intensive care unit (ICU) [2]. HF and renal injury are intricately connected through a complex web of organ interactions. In its most severe form, this cardio-renal dysregulation leads to a phenomenon known as “cardio-renal syndrome,” encompassing a spectrum of acute or chronic heart and kidney disorders characterized by mutual deterioration [3, 4]. Given the consistent association between AKI and increased mortality in HF patients, it is crucial to identify HF patients at high risk of AKI in the intensive care unit (ICU) to improve their prognosis.

Previous research has identified certain clinical biomarkers, including elevated B-type natriuretic peptide (BNP), cystatin C (CysC), ST2, and albuminuria, which are associated with AKI in HF [5]. However, there is a limited number of well-established biomarkers for critically ill patients who experience HF. Therefore, there is a need to further explore appropriate risk stratification for AKI in critically ill HF patients and personalize their care.

Insulin resistance (IR), characterized by reduced effectiveness of insulin in the promotion of glucose uptake and utilization, plays a vital role in the development of HF and the deterioration of renal function [6]. The triglyceride-glucose (TyG) index, calculated using fasting triglyceride (TG) and fasting plasma glucose (FPG) levels, has become a straightforward surrogate marker for IR [7]. Multiple investigations have demonstrated a positive association between elevated TyG index values and the incidence rates of hypertension, HF, coronary artery disease, and chronic kidney disease (CKD) [8–12]. Moreover, the TyG index has demonstrated its reliability and convenience as a prognostic indicator for adverse outcomes in patients with kidney disease [13, 14].

An elevated TyG index has consistently shown a strong correlation with the incidence of AKI in many other critical scenarios, such as contrast-induced AKI [15]. Nevertheless, the clinical assessment of the TyG index among patients experiencing severe HF afflicted with AKI remains inadequately addressed within the existing literature. Therefore, we undertook a retrospective cohort study aiming to examine the prognostic value of the TyG index for AKI in critically ill patients diagnosed with HF.

## Methods

### Data selection

This study employed a retrospective observational design, utilizing data from the publicly available Medical Information Mart for Intensive Care IV (MIMIC-IV) database (https://mimic.mit.edu), specifically the records of ICU patients at the Beth Israel Deaconess Medical Center between the years 2008 and 2019 [16]. In order to comply with relevant regulations, the author Zewen Yang obtained both a Collaborative Institutional Training Initiative (CITI) license, along with the necessary permissions to utilize the MIMIC-IV database. The study was reported according to the STROCSS guidelines.

This study focused on a study population in MIMIC-IV consisting of 6,697 individuals who were diagnosed with HF and non-consecutively admitted to the ICU (≥ 18 years of age). In cases where patients had multiple admissions, only their initial stay was considered. To ensure data integrity, patients were excluded if they lacked AKI data within 48 h of ICU admission, had incomplete information on triglyceride (TG) and glucose levels, or lacked follow-up data.

**Fig. 1:**
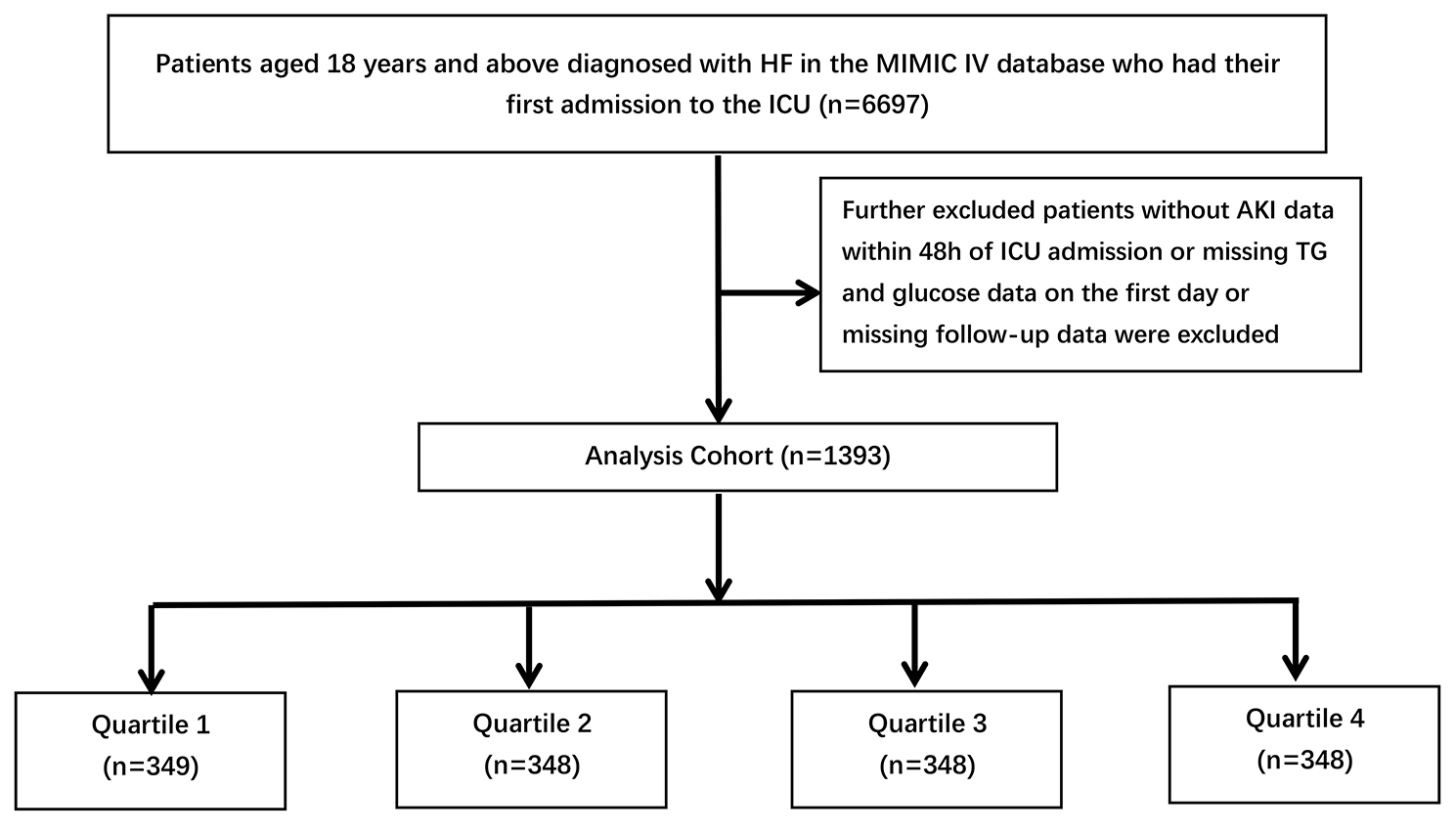
Flow chart of patient selection

Ultimately, a final study cohort of 1,393 patients was established and divided into four groups based on the quartiles of the TyG index observed on the first day of their ICU stay (Fig. 1).

### Data collection

Data collection involved the utilization of Structured Query Language (SQL) with PostgreSQL (version 14.2) to extract baseline characteristics of patients. These characteristics encompassed patient demographics (age, gender, body mass index (BMI) and race), vital signs (heart rate (HR), systolic blood pressure (SBP), and diastolic blood pressure (DBP)), severity at admission (measured by Simplified acute physiological score II (SAPSII), Systemic inflammatory response syndrome (SIRS) score, Acute physiology score III (APSIII), and the Sequential Organ Failure Assessment (SOFA) score), medication details (including beta blocker, loop diuretics (including furosemide and torsemide), angiotensin converting enzyme inhibitor (ACEI), angiotensin receptor blocker (ARB), angiotensin receptor-neprilysin inhibitor (ARNI), laboratory test results (red blood cell (RBC), white blood cells (WBC), neutrophils, hemoglobin, lymphocytes, platelets, albumin, urine creatinine (Ucr), serum creatinine (Scr), blood urea nitrogen (BUN), creatine kinase MB (CKMB), partial pressure of CO2 (PCO2), C-reactive protein (CRP), total cholesterol (TC), total glyceride (TG), high density lipoprotein cholesterol (HDL-C), NT-proBNP, low density lipoprotein cholesterol (LDL-C), potassium, sodium, pH, PO2, glucose, and HbA1c and TnT) within the initial 24 h of ICU admission, as well as information on comorbidities obtain from the MIMIC-IV database.

The TyG index was calculated using the following formula: ln [fasting TG (mg/dl) ×fasting glucose (mg/dl)]/2.

Chronic obstructive pulmonary disease (COPD), dyslipidemia, hypertension, atrial fibrillation (AF), diabetes, percutaneous coronary intervention (PCI), acute myocardial infarction (AMI) coronary artery bypass grafting (CABG), and CKD were defined using International Classification of Diseases, 10th Revision (ICD-10) and ICD-9 codes. The follow-up period commenced on the admission date and concluded when the endpoints of interest occured.

To address missing values, the researchers employed the multiple imputation (*missForest R*) approach. Variables with missing rates exceeding 25% were transformed into dummy variables in the models to mitigate potential bias that could arise from directly imputing the missing values. BMI, SBP, DBP, CKMB, CRP, HbA1c, Ucr, HDL-C, LDL-C, lymphocytes, neutrophils, NT-proBNP, TC and TnT contained>25% missing value.

### Endpoints of interest

The primary endpoint was the incidence of AKI. AKI was defined in accordance with the Kidney Disease: Improving Global Outcomes (KDIGO) guidelines. This involved an increase in SCr to ≥ 1.5 times the baseline within the prior 7 days; or a rise of ≥ 0.3 mg/dl in SCr within 48 h; or urine volume of < 0.5 ml/kg/h for 6 h or more [17]. Minimum of the SCr values available within the 7 days before admission was used as the baseline SCr [18, 19]. In cases where pre-admission SCr values were unavailable, the initial SCr measurement at admission was used as the baseline.

The secondary endpoint encompassed in-hospital mortality for both the whole study population and the AKI subset. The use of the renal replacement therapy (RRT) which represented the progression of AKI severity was also included as a secondary endpoint representing renal outcome.

### Statistical analysis

Continuous variables were described using either the mean (standard deviation (SD)) or median (interquartile range (IQR)), and comparisons between groups were made using the Mann–Whitney U test or the student t-test, depending on the data’s nature. Categorical variables were expressed as frequencies and percentages (%) and were compared between groups using either the Fisher’s exact test or Pearson chi-square test.

The Kaplan-Meier survival analysis was employed to estimate the incidence of AKI, the use of RRT and the in-hospital mortality among groups based on the TyG index.

Cox proportional hazards models were utilized to compute the hazard ratio (HR) and the 95% confidence interval (CI) for the TyG index and incidence of AKI between groups and adjusted for multiple variables. Model 1 represented an unadjusted analysis, while Model 2 involved adjustments for sex, age, and BMI. Model 3 incorporated variables from Model 2 and further adjusted for additional factors such as race, SBP, DBP, HR, SOFA, SIRS, APSIII, SAPSII, digoxin, beta blocker, ACEI, ARB, ARNI, loop diuretics, WBC, RBC, hemoglobin, neutrophils, lymphocytes, platelets, albumin, BUN, Scr, Ucr, CKMB, PCO2, LDL-C, HDL-C, TG, CRP, NT-proBNP, potassium, sodium, pH, PO2, HbA1c and TnT. The TyG index was incorporated into the models in both continuous and categorical forms. HRs were calculated, and the findings were presented with 95% confidence intervals CIs. The lowest quartile of the TyG index was used as the baseline group in all four models.

Moreover, a restricted cubic splines model was employed to investigate the potential dose-response association between the TyG index and the incidence of AKI, and adjusted for multiple models as mentioned above.

Subgroup analyses were performed to explore the consistency of the prognostic value of the TyG index within different subgroups. These subgroups were defined based on age (< 65 versus ≥ 65 years), sex (female versus male), BMI (< 30 versus ≥ 30 kg/m^2^), and the presence of specific medical histories such as diabetes, CKD, AMI, and hypertension. Likelihood ratio tests were used to assess the relationship between TyG index and variables used for stratification.

All data analyses were conducted using R version 4.2.2 (R Foundation for Statistical Computing, Vienna, Austria). A two-sided p-value was below 0.05.

## Results

In total, 1,393 patients were enrolled in this study. Their median age was 71.00 [60.00, 81.00] years, and 822 (59%) were male. The median TyG index value was 4.82 [4.61, 5.07]. The incidence of AKI was 82.8%.

### Baseline characteristics

Table 1 presents the baseline characteristics of the patients divided into quartiles based on the TyG index (quartile Q1: 3.61–4.61; Q2: 4.61–4.82; Q3: 4.82–5.07; Q4: 5.07–7.18). The median TyG index of the four groups were 4.47 [4.37, 4.55], 4.71 [4.67, 4.78], 4.94 [4.88, 5.00], and 5.28 [5.17, 5.48], respectively. Among the patients in the Q4 group, a younger age and higher scores of illness severity at admission were observed. Additionally, they exhibited a lower incidence of AF, but a higher incidence of AMI, PCI, CABG, and diabetes. Furthermore, this group demonstrated higher levels of blood urea nitrogen (BUN), glucose, platelets, potassium, Scr, TG, WBC, as well as lower levels of albumin, PH, and sodium. They also had a lower frequency of ACEI and digoxin use, compared with the lower TyG index group (all P < 0.05). Moreover, patients with BMI greater than 30 kg/m^2^, values of HbA1c greater than 6.4%, values of LDL-C greater than 103 mg/dl, values of HDL-C less than 33 mg/dl, percentage of lymphocytes between 7 and 11.50%, percentage of neutrophils between 79.60 and 85.90% were more common in Q4 group (all P < 0.05). With a higher TyG index, there was a gradual increase in the incidence of AKI (79.4% vs. 82.5% vs. 84.5% vs. 85.1%, P = 0.183).

**Table 1:** Baseline characteristics according to TyG index quartiles^a^

Variables	Overall	Q1	Q2	Q3	Q4	P value
Number of patients	1393	349	348	348	348	
Male,n(%)	822 (59.0)	208 (59.6)	204 (58.6)	211 (60.6)	199 (57.2)	0.818
Age,years,(median [IQR])	71.00 [60.00, 81.00]	75.00 [63.00, 84.00]	73.50 [61.75, 82.00]	70.00 [59.75, 80.00]	66.00 [57.00, 75.00]	< 0.001
Race,n(%)						0.069
White	915 (65.7)	222 (63.6)	230 (66.1)	232 (66.7)	231 (66.4)	
Black	97 (7.0)	38 (10.9)	22 (6.3)	20 (5.7)	17 (4.9)	
Asian	32 (2.3)	11 (3.2)	9 (2.6)	4 (1.1)	8 (2.3)	
Others	349 (25.1)	78 (22.3)	87 (25.0)	92 (26.4)	92 (26.4)	
BMI,kg/m2,n(%)						0.001
<30	415 (29.8)	117 (33.5)	114 (32.8)	97 (27.9)	87 (25.0)	
>=30	244 (17.5)	47 (13.5)	57 (16.4)	54 (15.5)	86 (24.7)	
Missing	734 (52.7)	185 (53.0)	177 (50.9)	197 (56.6)	175 (50.3)	
ASPIII (median [IQR])	43.00 [32.00, 55.00]	41.00 [31.00, 52.00]	42.00 [32.00, 54.25]	43.00 [33.00, 55.00]	49.00 [35.00, 62.00]	< 0.001
SAPIII (median [IQR])	36.00 [29.00, 45.00]	35.00 [28.00, 43.00]	35.00 [30.00, 44.00]	37.00 [29.00, 46.00]	37.50 [29.00, 47.00]	0.11
SIRS score (median [IQR])	3.00 [2.00, 3.00]	2.00 [2.00, 3.00]	2.00 [2.00, 3.00]	3.00 [2.00, 3.00]	3.00 [2.00, 3.00]	< 0.001
SOFA score (median [IQR])	4.00 [2.00, 7.00]	4.00 [2.00, 7.00]	4.00 [2.00, 7.00]	4.00 [2.00, 7.00]	5.00 [3.00, 8.00]	< 0.001
Vital Signs						
HR (median [IQR])	87.00 [75.00, 101.00]	85.00 [75.00, 100.00]	87.00 [75.00, 102.25]	87.00 [75.00, 99.00]	88.00 [78.00, 103.00]	0.183
SBP,mmhg,n(%)						0.268
< 90	13 (0.9)	4 (1.1)	3 (0.9)	4 (1.1)	2 (0.6)	
>140	158 (11.3)	37 (10.6)	32 (9.2)	37 (10.6)	52 (14.9)	
90–140	496 (35.6)	124 (35.5)	124 (35.6)	115 (33.0)	133 (38.2)	
Missing	726 (52.1)	184 (52.7)	189 (54.3)	192 (55.2)	161 (46.3)	
DBP,mmhg,n(%)						0.367
< 60	94 (6.7)	28 (8.0)	24 (6.9)	19 (5.5)	23 (6.6)	
> 90	53 (3.8)	13 (3.7)	11 (3.2)	15 (4.3)	14 (4.0)	
60–90	520 (37.3)	124 (35.5)	124 (35.6)	122 (35.1)	150 (43.1)	
Missing	726 (52.1)	184 (52.7)	189 (54.3)	192 (55.2)	161 (46.3)	
Laboratory tests						
Lymphocyte,%,n(%)						< 0.001
<7	281 (20.2)	49 (14.0)	74 (21.3)	76 (21.8)	82 (23.6)	
> 18.8	202 (14.5)	51 (14.6)	42 (12.1)	49 (14.1)	60 (17.2)	
11.50–18.80	253 (18.2)	74 (21.2)	63 (18.1)	65 (18.7)	51 (14.7)	
7–11.50	274 (19.7)	54 (15.5)	59 (17.0)	72 (20.7)	89 (25.6)	
Missing	383 (27.5)	121 (34.7)	110 (31.6)	86 (24.7)	66 (19.0)	
Neutrophils,%,n(%)						< 0.001
<69.8	211 (15.1)	58 (16.6)	50 (14.4)	46 (13.2)	57 (16.4)	
> 85.9	277 (19.9)	53 (15.2)	67 (19.3)	76 (21.8)	81 (23.3)	
69.8–79.60	251 (18.0)	68 (19.5)	55 (15.8)	71 (20.4)	57 (16.4)	
79.60–85.90	271 (19.5)	49 (14.0)	66 (19.0)	69 (19.8)	87 (25.0)	
Missing	383 (27.5)	121 (34.7)	110 (31.6)	86 (24.7)	66 (19.0)	
Platelets,(K/uL),(median [IQR])	209.00 [161.00, 268.00]	198.00 [151.00, 254.00]	208.00 [165.00, 262.00]	212.00 [164.00, 273.50]	214.50 [165.75, 282.00]	0.03
WBC (median [IQR])	10.30 [7.60, 13.80]	8.40 [6.70, 11.40]	9.80 [7.70, 12.65]	11.60 [8.15, 14.60]	11.75 [8.60, 16.50]	< 0.001
RBC,m/uL,(median [IQR])	3.92 [3.41, 4.43]	3.91 [3.47, 4.40]	3.95 [3.41, 4.41]	3.96 [3.42, 4.44]	3.86 [3.35, 4.44]	0.825
Hemoglobin,g/dL,(median [IQR])	11.70 [10.10, 13.20]	11.70 [10.40, 13.20]	11.70 [10.10, 13.28]	11.80 [10.25, 13.25]	11.30 [9.80, 13.20]	0.312
PCO2,mmhg,(median [IQR])	41.00 [35.00, 48.00]	40.00 [34.00, 46.00]	40.00 [34.00, 48.00]	41.00 [35.50, 49.00]	41.00 [36.00, 49.00]	0.029
PH (median [IQR])	7.39 [7.33, 7.44]	7.41 [7.35, 7.44]	7.40 [7.36, 7.45]	7.39 [7.33, 7.44]	7.37 [7.30, 7.42]	< 0.001
PO2,mmhg,(median [IQR])	99.00 [63.25, 190.75]	102.00 [66.00, 241.00]	98.50 [67.00, 199.75]	89.00 [56.00, 161.50]	104.50 [65.75, 187.25]	0.073
Albumin,g/dL(median [IQR])	3.30 [2.80, 3.70]	3.50 [3.00, 3.80]	3.40 [2.90, 3.70]	3.20 [2.80, 3.70]	3.20 [2.70, 3.60]	< 0.001
BUN,mg/dL(median [IQR])	22.00 [16.00, 35.00]	21.00 [16.00, 31.00]	21.00 [16.00, 34.25]	23.00 [17.00, 37.00]	23.00 [16.00, 40.00]	0.022
SCR, mg/dL,(median [IQR])	1.10 [0.80, 1.50]	1.00 [0.80, 1.40]	1.00 [0.80, 1.40]	1.10 [0.90, 1.63]	1.10 [0.90, 1.80]	0.001
UCR,mg/dL,n(%)						0.058
< 48	134 (9.6)	35 (10.0)	29 (8.3)	28 (8.0)	42 (12.1)	
> 122	135 (9.7)	30 (8.6)	30 (8.6)	42 (12.1)	33 (9.5)	
48–78	127 (9.1)	33 (9.5)	24 (6.9)	38 (10.9)	32 (9.2)	
78–122	132 (9.5)	22 (6.3)	33 (9.5)	43 (12.4)	34 (9.8)	
Missing	865 (62.1)	229 (65.6)	232 (66.7)	197 (56.6)	207 (59.5)	
HDL,mg/dL,n(%)						< 0.001
< 33	248 (17.8)	40 (11.5)	67 (19.3)	71 (20.4)	70 (20.1)	
> 53	238 (17.1)	98 (28.1)	71 (20.4)	40 (11.5)	29 (8.3)	
33–42	272 (19.5)	64 (18.3)	74 (21.3)	72 (20.7)	62 (17.8)	
42–53	258 (18.5)	87 (24.9)	61 (17.5)	61 (17.5)	49 (14.1)	
Missing	377 (27.1)	60 (17.2)	75 (21.6)	104 (29.9)	138 (39.7)	
LDL,mg/dL,n(%)						< 0.001
< 55	249 (17.9)	80 (22.9)	65 (18.7)	55 (15.8)	49 (14.1)	
> 103	245 (17.6)	52 (14.9)	58 (16.7)	74 (21.3)	61 (17.5)	
55–76	255 (18.3)	85 (24.4)	67 (19.3)	62 (17.8)	41 (11.8)	
76–103	250 (17.9)	71 (20.3)	82 (23.6)	53 (15.2)	44 (12.6)	
Missing	394 (28.3)	61 (17.5)	76 (21.8)	104 (29.9)	153 (44.0)	
TG,mg/dL,(median [IQR])	113.00 [84.00, 164.00]	70.00 [57.00, 85.00]	104.00 [86.00, 119.25]	138.00 [108.00, 168.25]	209.50 [154.00, 290.25]	< 0.001
TC,mg/dL,n(%)						< 0.001
<116	257 (18.4)	89 (25.5)	70 (20.1)	56 (16.1)	42 (12.1)	
>174	259 (18.6)	51 (14.6)	59 (17.0)	68 (19.5)	81 (23.3)	
116–145	275 (19.7)	92 (26.4)	77 (22.1)	59 (17.0)	47 (13.5)	
145–174	253 (18.2)	60 (17.2)	72 (20.7)	70 (20.1)	51 (14.7)	
Missing	349 (25.1)	57 (16.3)	70 (20.1)	95 (27.3)	127 (36.5)	
Glucose,mg/dL,(median [IQR])	129.00 [106.00, 172.00]	104.00 [92.00, 120.00]	121.50 [104.00, 143.00]	141.50 [114.00, 173.00]	202.00 [147.00, 263.25]	< 0.001
HbA1c%,n(%)						< 0.001
< 5.7	275 (19.7)	98 (28.1)	86 (24.7)	61 (17.5)	30 (8.6)	
> 6.4	261 (18.7)	27 (7.7)	53 (15.2)	62 (17.8)	119 (34.2)	
5.7–6.4	287 (20.6)	93 (26.6)	78 (22.4)	67 (19.3)	49 (14.1)	
Missing	570 (40.9)	131 (37.5)	131 (37.6)	158 (45.4)	150 (43.1)	
TNTµg/L,n(%)						0.001
<0.06	202 (14.5)	60 (17.2)	51 (14.7)	49 (14.1)	42 (12.1)	
>0.975	203 (14.6)	34 (9.7)	45 (12.9)	54 (15.5)	70 (20.1)	
0.06–0.25	208 (14.9)	45 (12.9)	55 (15.8)	52 (14.9)	56 (16.1)	
0.25–0.975	198 (14.2)	36 (10.3)	61 (17.5)	56 (16.1)	45 (12.9)	
Missing	582 (41.8)	174 (49.9)	136 (39.1)	137 (39.4)	135 (38.8)	
CKMB(IU/L),n(%)						0.299
< 3	180 (12.9)	49 (14.0)	46 (13.2)	45 (12.9)	40 (11.5)	
> 18	280 (20.1)	66 (18.9)	55 (15.8)	84 (24.1)	75 (21.6)	
3–6	2 (0.1)	0 (0.0)	1 (0.3)	0 (0.0)	1 (0.3)	
6–18	13 (0.9)	2 (0.6)	6 (1.7)	2 (0.6)	3 (0.9)	
Missing	918 (65.9)	232 (66.5)	240 (69.0)	217 (62.4)	229 (65.8)	
NT-proBNP,pg/mL,n(%)						0.27
<1632	78 (5.6)	21 (6.0)	16 (4.6)	14 (4.0)	27 (7.8)	
>10,521	78 (5.6)	20 (5.7)	17 (4.9)	23 (6.6)	18 (5.2)	
1632–3955	78 (5.6)	14 (4.0)	20 (5.7)	21 (6.0)	23 (6.6)	
3955–10521	77 (5.5)	13 (3.7)	26 (7.5)	22 (6.3)	16 (4.6)	
Missing	1082 (77.7)	281 (80.5)	269 (77.3)	268 (77.0)	264 (75.9)	
CRP(mg/L),n(%)						0.295
< 13.95	41 (2.9)	15 (4.3)	13 (3.7)	8 (2.3)	5 (1.4)	
>134.4	41 (2.9)	7 (2.0)	9 (2.6)	12 (3.4)	13 (3.7)	
13.95–57.90	41 (2.9)	9 (2.6)	10 (2.9)	14 (4.0)	8 (2.3)	
57.90-134.4	40 (2.9)	6 (1.7)	14 (4.0)	11 (3.2)	9 (2.6)	
Missing	1230 (88.3)	312 (89.4)	302 (86.8)	303 (87.1)	313 (89.9)	
Potassium,mmol/L,(median [IQR])	4.10 [3.80, 4.50]	4.10 [3.70, 4.50]	4.10 [3.80, 4.50]	4.10 [3.80, 4.50]	4.20 [3.80, 4.70]	0.03
Sodium,mmol/L,(median [IQR])	139.00 [136.00, 141.00]	140.00 [136.00, 142.00]	139.00 [136.00, 142.00]	139.00 [136.00, 141.00]	138.00 [135.00, 141.00]	< 0.001
TyG index,(median [IQR])	4.82 [4.61, 5.07]	4.47 [4.37, 4.55]	4.71 [4.67, 4.78]	4.94 [4.88, 5.00]	5.28 [5.17, 5.48]	< 0.001
Comorbidities,n (%)						
AF,n(%)	613 (44.0)	185 (53.0)	165 (47.4)	132 (37.9)	131 (37.6)	< 0.001
AMI,n(%)	485 (34.8)	82 (23.5)	122 (35.1)	137 (39.4)	144 (41.4)	< 0.001
CKD,n(%)	383 (27.5)	96 (27.5)	89 (25.6)	96 (27.6)	102 (29.3)	0.748
COPD,n(%)	164 (11.8)	41 (11.7)	47 (13.5)	47 (13.5)	29 (8.3)	0.113
Dyslipidemia,n(%)	658 (47.2)	156 (44.7)	168 (48.3)	159 (45.7)	175 (50.3)	0.443
Diabetes,n(%)	524 (37.6)	65 (18.6)	105 (30.2)	135 (38.8)	219 (62.9)	< 0.001
Hypertension,n(%)	494 (35.5)	130 (37.2)	121 (34.8)	123 (35.3)	120 (34.5)	0.871
PCI,n(%)	189 (13.6)	29 (8.3)	45 (12.9)	55 (15.8)	60 (17.2)	0.003
CABG,n(%)	69 (5.0)	14 (4.0)	11 (3.2)	17 (4.9)	27 (7.8)	0.031
Medications						
ARNI,n(%)	11 (0.8)	2 (0.6)	4 (1.1)	3 (0.9)	2 (0.6)	0.798
ACEI,n(%)	634 (45.5)	164 (47.0)	164 (47.1)	172 (49.4)	134 (38.5)	0.021
ARB,n(%)	135 (9.7)	25 (7.2)	40 (11.5)	38 (10.9)	32 (9.2)	0.209
Beta blocker,n(%)	1090 (78.2)	267 (76.5)	276 (79.3)	287 (82.5)	260 (74.7)	0.07
Digoxin, n(%)	165 (11.8)	48 (13.8)	53 (15.2)	34 (9.8)	30 (8.6)	0.019
Loop diuretics,n(%),n(%)	1129 (81.0)	277 (79.4)	284 (81.6)	282 (81.0)	286 (82.2)	0.8
Events						
AKI^b^,n(%)	1154 (82.8)	277 (79.4)	287 (82.5)	294 (84.5)	296 (85.1)	0.183

ACEI (angiotensin-converting-enzyme inhibitors), AKI (acute kidney injury), AMI (acute myocardial infarction), APSIII (acute physiology score III), ARB (angiotensin receptor blocker), angiotensin receptor-neprilysin inhibitor (ARNI), BUN (blood urea nitrogen), BMI (body mass index), CABG (coronary artery bypass grafting), CKD (chronic kidney disease), COPD (chronic obstructive pulmonary disease), HDL (high-density lipoprotein), HbA1c (hemoglobin A1c), LDL (low-density lipoprotein), PCI (percutaneous coronary intervention), RBC (red blood cell), Scr (serum creatinine), SAPSII (simplified acute physiological score II), SIRS (systemic inflammatory response syndrome), SOFA (sequential organ failure assessment), TC (total cholesterol), TG (triglyceride), TyG index (triglyceride glucose index), Ucr (urine creatinine), WBC (white blood cell)

a TyG index: Q1: 3.61–4.61; Q2: 4.61–4.82; Q3: 4.82–5.07; Q4: 5.07–7.18;

b AKI was defined in accordance with Kidney Disease: Improving Global Outcomes (KDIGO) guidelines as an increase in SCr to ≥ 1.5 times baseline must have occurred within the prior 7 days; or a ≥ 0.3 mg/dl increase in SCr occurred within 48 h; or urine volume < 0.5 ml/kg/h for 6 h or more

Table 2 displays the baseline characteristics comparing AKI patients to non-AKI patients. The AKI group had a higher proportion of male patients, tended to have a BMI below 30 kg/m^2^, and showed a higher incidence of AF, CKD, hypertension, and greater use of loop diuretics. In terms of laboratory indicators, AKI patients had higher levels of BUN, potassium, Scr, TG, and CKMB, but lower levels of albumin (all P < 0.05). Notably, the AKI group had a higher incidence of TC levels below 116 mg/dl, LDL-C levels below 55 mg/dl, HDL-C levels below 33 mg/dl, lymphocyte percentages below 7%, UCR levels above 122 mg/dl, neutrophil percentages above 85.9% (all P < 0.05). SIRS scores, SOFA scores, APSIII, and SAPSII were also higher in the AKI group compared with the non-AKI group (all P < 0.05). The AKI group exhibited a significantly higher TyG index than the non-AKI group (4.83 [4.63, 5.08] vs. 4.78 [4.57, 5.01], P = 0.016).

**Table 2:** Baseline characteristics of the AKI and Non-AKI groups

Variables	Overall	non-AKI	AKI	p value
Number of patients	1393	239	1154	
Male,n(%)	822 (59.0)	123 (51.5)	699 (60.6)	0.011
Age,years,(median [IQR])	71.00 [60.00, 81.00]	71.00 [59.00, 82.00]	71.00 [60.00, 81.00]	0.417
Race,n(%)				0.1
White	915 (65.7)	156 (65.3)	759 (65.8)	
Black	97 (7.0)	20 (8.4)	77 (6.7)	
Asian	32 (2.3)	10 (4.2)	22 (1.9)	
Others	349 (25.1)	53 (22.2)	296 (25.6)	
BMI,kg/m2,n(%)				0.037
<30	415 (29.8)	87 (36.4)	328 (28.4)	
>=30	244 (17.5)	34 (14.2)	210 (18.2)	
Missing	734 (52.7)	118 (49.4)	616 (53.4)	
ASPIII (median [IQR])	43.00 [32.00, 55.00]	34.00 [26.00, 45.00]	45.00 [34.00, 58.00]	< 0.001
SAPIII (median [IQR])	36.00 [29.00, 45.00]	31.00 [24.50, 37.00]	37.00 [30.00, 46.00]	< 0.001
SIRS score (median [IQR])	3.00 [2.00, 3.00]	2.00 [2.00, 3.00]	3.00 [2.00, 3.00]	0.02
SOFA score (median [IQR])	4.00 [2.00, 7.00]	3.00 [1.00, 4.50]	5.00 [3.00, 8.00]	< 0.001
Vital Signs				
HR (median [IQR])	87.00 [75.00, 101.00]	86.00 [74.50, 101.50]	87.00 [76.00, 101.00]	0.403
SBP,mmhg,n(%)				0.714
< 90	13 (0.9)	1 (0.4)	12 (1.0)	
>140	158 (11.3)	30 (12.6)	128 (11.1)	
90–140	496 (35.6)	87 (36.4)	409 (35.4)	
Missing	726 (52.1)	121 (50.6)	605 (52.4)	
DBP,mmhg,n(%)				0.768
< 60	94 (6.7)	14 (5.9)	80 (6.9)	
> 90	53 (3.8)	11 (4.6)	42 (3.6)	
60–90	520 (37.3)	93 (38.9)	427 (37.0)	
Missing	726 (52.1)	121 (50.6)	605 (52.4)	
Laboratory tests				
Lymphocyte,%,n(%)				< 0.001
<7	281 (20.2)	32 (13.4)	249 (21.6)	
> 18.8	202 (14.5)	40 (16.7)	162 (14.0)	
11.50–18.80	253 (18.2)	42 (17.6)	211 (18.3)	
7–11.50	274 (19.7)	28 (11.7)	246 (21.3)	
Missing	383 (27.5)	97 (40.6)	286 (24.8)	
Neutrophils,%,n(%)				< 0.001
<69.8	211 (15.1)	41 (17.2)	170 (14.7)	
> 85.9	277 (19.9)	35 (14.6)	242 (21.0)	
69.8–79.60	251 (18.0)	33 (13.8)	218 (18.9)	
79.60–85.90	271 (19.5)	33 (13.8)	238 (20.6)	
Missing	383 (27.5)	97 (40.6)	286 (24.8)	
Platelets,(K/uL),(median [IQR])	209.00 [161.00, 268.00]	215.00 [167.00, 272.25]	208.00 [160.00, 268.00]	0.237
WBC (median [IQR])	10.30 [7.60, 13.80]	10.10 [7.30, 12.88]	10.40 [7.70, 14.00]	0.052
RBC,m/uL,(median [IQR])	3.92 [3.41, 4.43]	3.93 [3.40, 4.42]	3.91 [3.41, 4.43]	0.939
Hemoglobin,g/dL,(median [IQR])	11.70 [10.10, 13.20]	11.90 [10.20, 13.20]	11.60 [10.10, 13.30]	0.352
PCO2,mmhg,(median [IQR])	41.00 [35.00, 48.00]	41.00 [35.00, 48.00]	40.00 [35.00, 48.00]	0.721
PH (median [IQR])	7.39 [7.33, 7.44]	7.39 [7.34, 7.42]	7.39 [7.33, 7.44]	0.996
PO2,mmhg,(median [IQR])	41.00 [35.00, 48.00]	41.00 [35.00, 48.00]	40.00 [35.00, 48.00]	0.721
Albumin,g/dL(median [IQR])	3.30 [2.80, 3.70]	3.50 [3.10, 3.82]	3.30 [2.80, 3.70]	< 0.001
BUN,mg/dL(median [IQR])	22.00 [16.00, 35.00]	20.00 [15.00, 30.50]	22.00 [16.25, 36.00]	< 0.001
SCR, mg/dL,(median [IQR])	1.10 [0.80, 1.50]	1.00 [0.80, 1.30]	1.10 [0.80, 1.60]	< 0.001
UCR,mg/dL,n(%)				< 0.001
< 48	134 (9.6)	16 (6.7)	118 (10.2)	
> 122	135 (9.7)	11 (4.6)	124 (10.7)	
48–78	127 (9.1)	16 (6.7)	111 (9.6)	
78–122	132 (9.5)	9 (3.8)	123 (10.7)	
Missing	865 (62.1)	187 (78.2)	678 (58.8)	
HDL,mg/dL,n(%)				< 0.001
< 33	248 (17.8)	31 (13.0)	217 (18.8)	
> 53	238 (17.1)	63 (26.4)	175 (15.2)	
33–42	272 (19.5)	57 (23.8)	215 (18.6)	
42–53	258 (18.5)	51 (21.3)	207 (17.9)	
Missing	377 (27.1)	37 (15.5)	340 (29.5)	
LDL,mg/dL,n(%)				< 0.001
< 55	249 (17.9)	32 (13.4)	217 (18.8)	
> 103	245 (17.6)	54 (22.6)	191 (16.6)	
55–76	255 (18.3)	56 (23.4)	199 (17.2)	
76–103	250 (17.9)	58 (24.3)	192 (16.6)	
Missing	394 (28.3)	39 (16.3)	355 (30.8)	
TG,mg/dL,(median [IQR])	113.00 [84.00, 164.00]	105.00 [80.50, 155.50]	114.00 [84.25, 166.00]	0.046
TC,mg/dL,n(%)				< 0.001
<116	257 (18.4)	30 (12.6)	227 (19.7)	
>174	259 (18.6)	59 (24.7)	200 (17.3)	
116–145	275 (19.7)	62 (25.9)	213 (18.5)	
145–174	253 (18.2)	55 (23.0)	198 (17.2)	
Missing	349 (25.1)	33 (13.8)	316 (27.4)	
Glucose,mg/dL,(median [IQR])	129.00 [106.00, 172.00]	126.00 [103.00, 164.00]	130.00 [107.00, 173.00]	0.117
HbA1c%,n(%)				0.105
< 5.7	275 (19.7)	57 (23.8)	218 (18.9)	
> 6.4	261 (18.7)	46 (19.2)	215 (18.6)	
5.7–6.4	287 (20.6)	54 (22.6)	233 (20.2)	
Missing	570 (40.9)	82 (34.3)	488 (42.3)	
TNTµg/L,n(%)				0.286
<0.06	202 (14.5)	33 (13.8)	169 (14.6)	
>0.975	203 (14.6)	32 (13.4)	171 (14.8)	
0.06–0.25	208 (14.9)	27 (11.3)	181 (15.7)	
0.25–0.975	198 (14.2)	34 (14.2)	164 (14.2)	
Missing	582 (41.8)	113 (47.3)	469 (40.6)	
CKMB(IU/L),n(%)				< 0.001
< 3	180 (12.9)	33 (13.8)	147 (12.7)	
> 18	280 (20.1)	24 (10.0)	256 (22.2)	
3–6	2 (0.1)	0 (0.0)	2 (0.2)	
6–18	13 (0.9)	1 (0.4)	12 (1.0)	
Missing	918 (65.9)	181 (75.7)	737 (63.9)	
NT-proBNP,pg/mL,n(%)				0.401
<1632	78 (5.6)	17 (7.1)	61 (5.3)	
>10521	78 (5.6)	8 (3.3)	70 (6.1)	
1632–3955	78 (5.6)	15 (6.3)	63 (5.5)	
3955–10,521	77 (5.5)	13 (5.4)	64 (5.5)	
Missing	1082 (77.7)	186 (77.8)	896 (77.6)	
CRP(mg/L),n(%)				0.241
< 13.95	41 (2.9)	8 (3.3)	33 (2.9)	
>134.4	41 (2.9)	5 (2.1)	36 (3.1)	
13.95–57.90	41 (2.9)	6 (2.5)	35 (3.0)	
57.90–134.4	40 (2.9)	2 (0.8)	38 (3.3)	
Missing	1230 (88.3)	218 (91.2)	1012 (87.7)	
Potassium,mmol/L,(median [IQR])	4.10 [3.80, 4.50]	4.00 [3.70, 4.40]	4.20 [3.80, 4.60]	0.016
Sodium,mmol/L,(median [IQR])	139.00 [136.00, 141.00]	139.00 [136.00, 141.00]	139.00 [136.00, 142.00]	0.69
TyG index,(median [IQR])	4.82 [4.61, 5.07]	4.78 [4.57, 5.01]	4.83 [4.63, 5.08]	0.016
Comorbidities,n (%)				
AF,n(%)	613 (44.0)	82 (34.3)	531 (46.0)	0.001
AMI,n(%)	485 (34.8)	80 (33.5)	405 (35.1)	0.686
CKD,n(%)	383 (27.5)	43 (18.0)	340 (29.5)	< 0.001
COPD,n(%)	164 (11.8)	26 (10.9)	138 (12.0)	0.718
Dyslipidemia,n(%)	658 (47.2)	117 (49.0)	541 (46.9)	0.608
Diabetes,n(%)	524 (37.6)	77 (32.2)	447 (38.7)	0.069
Hypertension,n(%)	494 (35.5)	103 (43.1)	391 (33.9)	0.008
PCI,n(%)	189 (13.6)	41 (17.2)	148 (12.8)	0.094
CABG,n(%)	69 (5.0)	10 (4.2)	59 (5.1)	0.661
Medications				
ARNI,n(%)	11 (0.8)	1 (0.4)	10 (0.9)	0.756
ACEI,n(%)	634 (45.5)	120 (50.2)	514 (44.5)	0.126
ARB,n(%)	135 (9.7)	28 (11.7)	107 (9.3)	0.297
Beta blocker,n(%)	1090 (78.2)	183 (76.6)	907 (78.6)	0.545
Digoxin ,n(%)	165 (11.8)	22 (9.2)	143 (12.4)	0.201
Loop diuretics,n(%)	1129 (81.0)	154 (64.4)	975 (84.5)	< 0.001

ACEI (angiotensin-converting-enzyme inhibitors), AF (atrial fibrillation), AKI (acute kidney injury), AMI (acute myocardial infarction), APSIII (acute physiology score III), ARB (angiotensin receptor blocker), angiotensin receptor-neprilysin inhibitor (ARNI), BUN (blood urea nitrogen), BMI (body mass index), CABG (coronary artery bypass grafting), CKD (chronic kidney disease), COPD (chronic obstructive pulmonary disease), HDL (high-density lipoprotein), HbA1c (hemoglobin A1c), LDL (low-density lipoprotein), PCI (percutaneous coronary intervention), RBC (red blood cell), SAPSII (simplified acute physiological score II), Scr (serum creatinine), SIRS (systemic inflammatory response syndrome), SOFA (sequential organ failure assessment), TC (total cholesterol), TG (triglyceride), TyG index (triglyceride glucose index), Ucr (urine creatinine), WBC (white blood cell)

### Primary endpoint

**Fig. 2:**
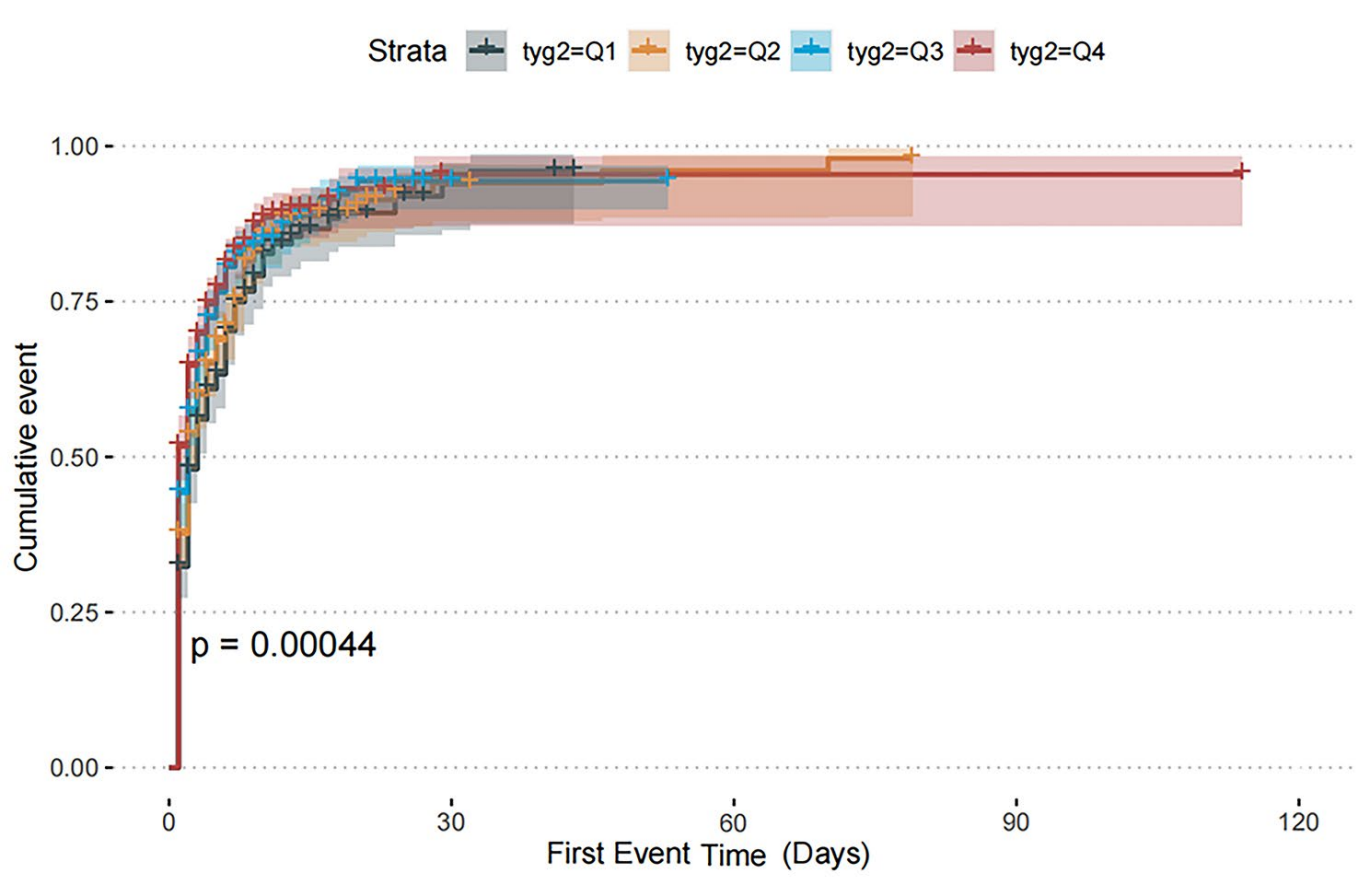
The cumulative event incidence curves for incidence of AKI. (TyG index quartile Q1: 3.61–4.61; Q2: 4.61–4.82; Q3: 4.82–5.07; Q4: 5.07–7.18)

Figure 2 presents the cumulative event incidence curve depicting the probability distribution of the incidence of AKI based on the TyG index quartiles. The incidence of AKI significantly differed among the groups during the period of follow-up (P<0.001).

When the TyG index was considered as a continuous variable, Cox proportional hazards analysis showed a statistically significant association between the risk of AKI and the TyG index in both unadjusted models (HR, 1.57 [95%CI 1.34–1.84]; P<0.001) and fully adjusted model (HR, 1.58 [95%CI 1.22–2.04]; P = 0.0006).

Furthermore, treating the TyG index as a nominal variable, the highest quartile (Q4) of the TyG index demonstrated a significant association with the risk of AKI in both the unadjusted model ( Q1 vs. Q2: HR, 1.11 [95% CI 0.94–1.31] P = 0.226; Q3: HR, 1.27 [95% CI 1.08–1.50] P = 0.004; Q4: HR, 1.42 [95% CI 1.20–1.67] P<0.001) and the fully adjusted model (Q1 vs. Q2: HR, 1.04 [95% CI 0.88–1.24] P = 0.655; Q3: HR, 1.16 [95% CI 0.96–1.39] P = 0.122; Q4: HR, 1.32 [95% CI 1.06–1.65] P = 0.012) (Table 3).

**Table 3:** Cox proportional hazard ratios (HR) for AKI incidence

Categories	Model 1		Model 2		Model 3	
	h (95% CI)	*P-*value	HR (95% CI)	*P-*value	HR (95% CI)	*P-*value
AKI incidence						
Continuous variable per 1 unit	1.57 [95% CI 1.34–1.84]	< 0.001	1.58 [95% CI 1.34–1.86]	< 0.001	1.58 [95%CI 1.22–2.04]	0.0006
Quartile^a^						
Q1(N = 349)	Ref.		Ref.		Ref.	
Q2(N = 348)	1.11 [95% CI 0.94–1.31]	0.226	1.10 [95% CI 0.93–1.30]	0.26	1.04 [95% CI 0.88–1.24]	0.655
Q3(N = 348)	1.27 [95% CI1.08–1.50]	0.004	1.24 [95% CI1.06–1.47]	0.009	1.16 [95% CI 0.96–1.39]	0.122
Q4(N = 348)	1.42 [95% CI 1.20–1.67]	<0.001	1.42 [95% CI1.20–1.67]	<0.001	1.32 [95% CI 1.06–1.65]	0.012

Model 1 was unadjusted

Model 2 was adjusted for sex, age, and BMI.

Model 3 was adjusted for the variables in model 2 and further adjusted for race, SBP, DBP, HR, SOFA, SIRS, APSIII, SAPSII, digoxin, beta blocker, ACEI, ARB, ARNI, loop diuretics, WBC, RBC, hemoglobin, neutrophils, lymphocytes, platelets, albumin, BUN, Scr, Ucr, CKMB, PCO2, CRP, TG, LDL-C, HDL-C, NT-proBNP, potassium, sodium, pH, PO2, HbA1c, TnT, AF, AMI, CKD, COPD, dyslipidemia, diabetes, hypertension, PCI and CABG.

^a^TyG index quartile Q1: 3.61–4.61; Q2: 4.61–4.82; Q3: 4.82–5.07; Q4: 5.07–7.18

**Fig. 3:**
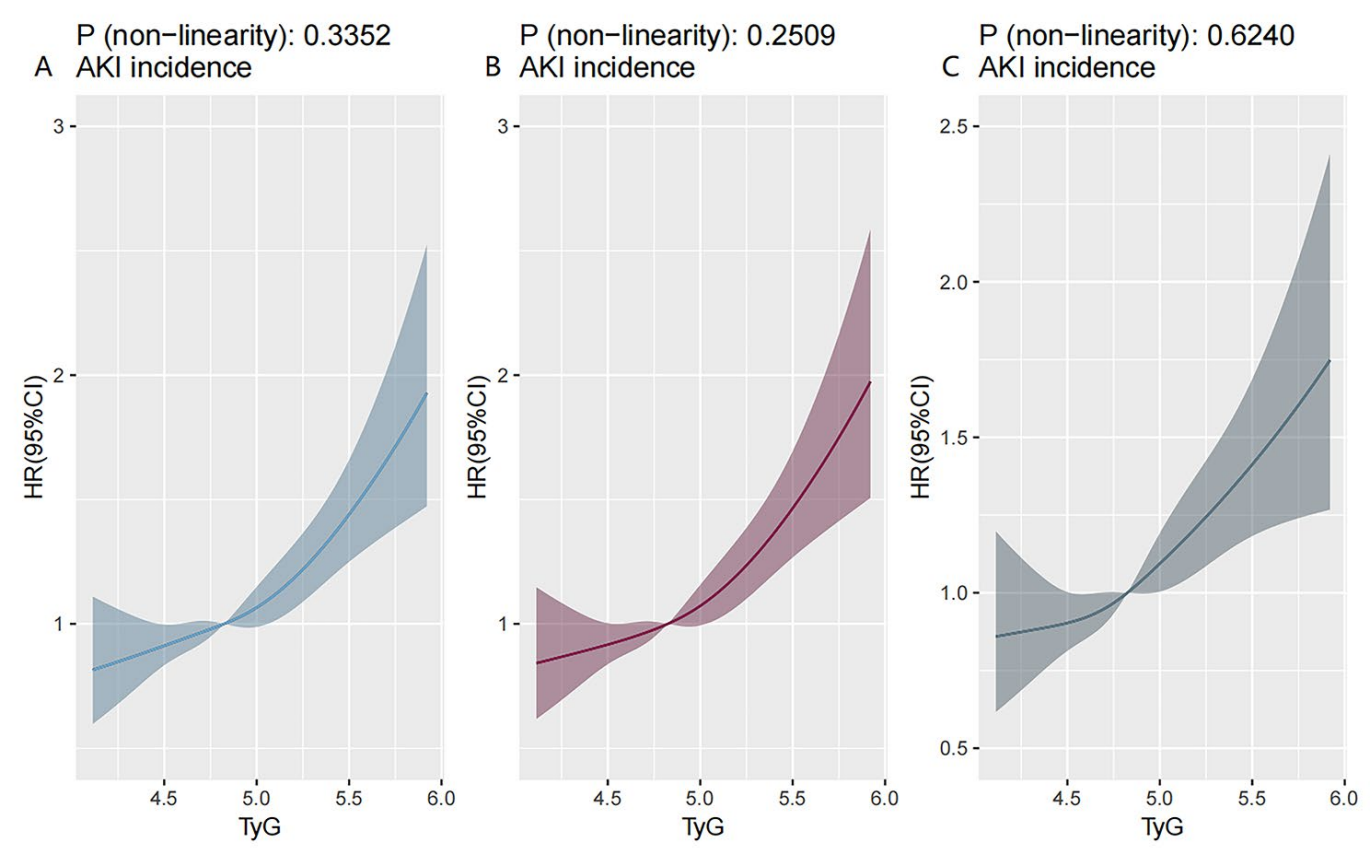
Restricted cubic spline curves for the TyG index hazard ratio. **A**: Model 1 was unadjusted. **B**: Model 2 was adjusted for age, sex, and BMI. **C**: Model 3 was adjusted for the variables in model 2 and further adjusted for race, SBP, DBP, HR, SOFA, SIRS, APSIII, SAPSII, digoxin, beta blocker, ACEI, ARB, ARNI, loop diuretics, WBC, RBC, hemoglobin, neutrophils, lymphocytes, platelets, albumin, BUN, Scr, Ucr, CKMB, PCO2, CRP, TG, LDL-C, HDL-C, NT-proBNP, potassium, sodium, pH, PO2, HbA1c and TnT.

Figure 3 shows the restricted cubic splines regression model, which demonstrated the dose-response relationship between the TyG index and AKI risk in both unadjusted and fully adjusted models (P for non-linearity = 0.335 and P for non-linearity = 0.624).

**Fig. 4:**
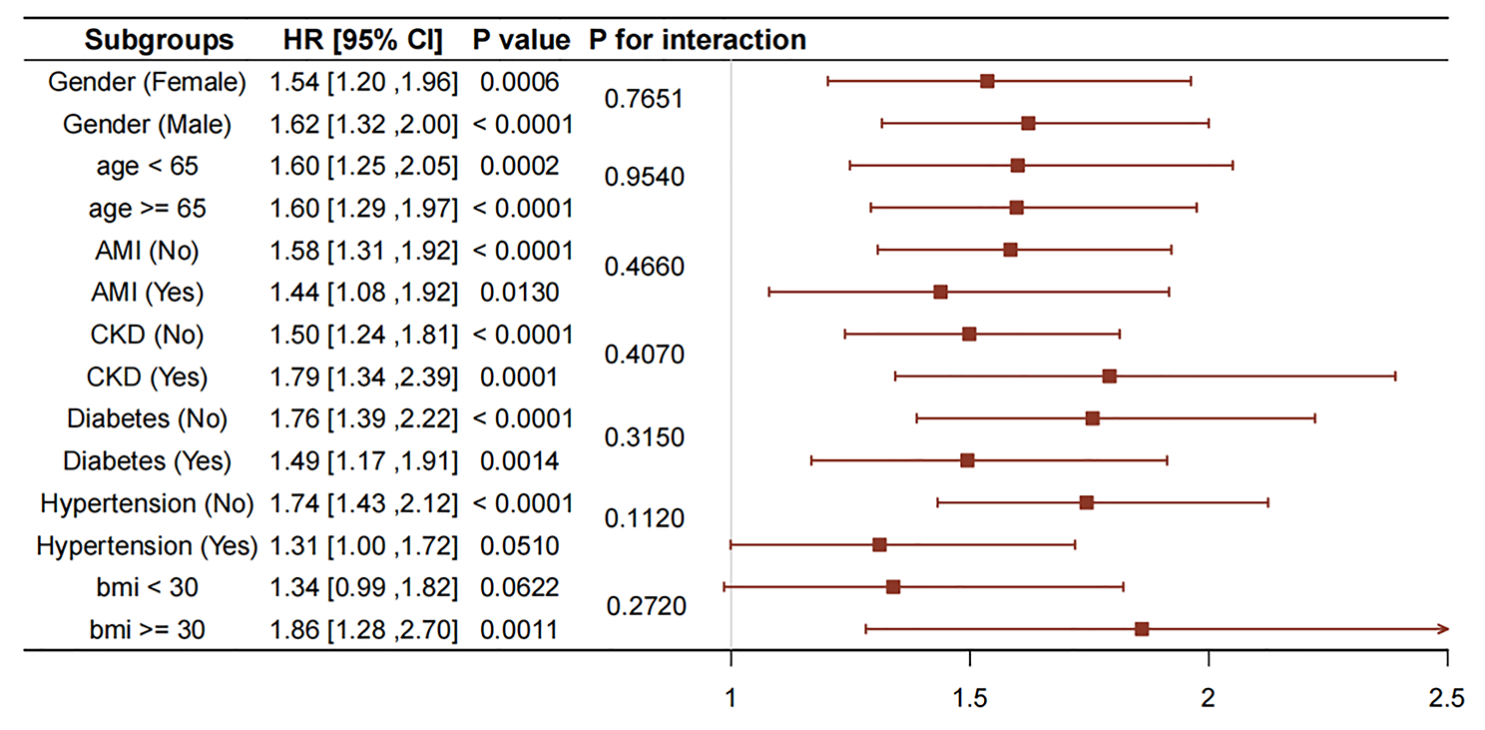
Forest plots of hazard ratios for the primary endpoint in different subgroups. AMI (acute myocardial infarction), BMI (body mass index), CKD (chronic kidney disease), CI (confidence interval), HR (hazard ratio)

Moreover, we conducted a risk stratification analysis of the TyG index for the primary endpoint in multiple subgroups, based on age, gender, AMI, CKD, hypertension, and diabetes (Fig. 4). The TyG index displayed a significant association with an increased risk of AKI in subgroups defined by female gender [HR (95% CI) 1.54 (1.20–1.96)], male gender [HR (95% CI) 1.62 (1.32-2.00)], age ≥ 65 years [HR (95% CI) 1.60 (1.29–1.97)], age<65 years [HR (95% CI) 1.60 (1.25–2.05)], BMI ≥ 30 kg/m^2^ [HR (95% CI) 1.86 (1.28–2.70)], presence of diabetes [HR (95% CI) 1.49 (1.17–1.91)], absence of diabetes [HR (95% CI) 1.76 (1.39–2.22)], absence of hypertension [HR (95% CI) 1.74(1.43–2.12)], absence of CKD [HR (95% CI) 1.50 (1.24, 1.81)], presence of CKD [HR (95% CI) 1.79 (1.34, 2.39)], presence of AMI [HR (95% CI) 1.44 (1.08, 1.92)], and absence of AMI [HR (95% CI) 1.58 (1.31, 1.92)] (all P < 0.05). Additionally, no relationships between the variables and the TyG index were observed in subgroup analyses (all p values for interaction > 0.05).

### Secondary endpoints

**Fig. 5:**
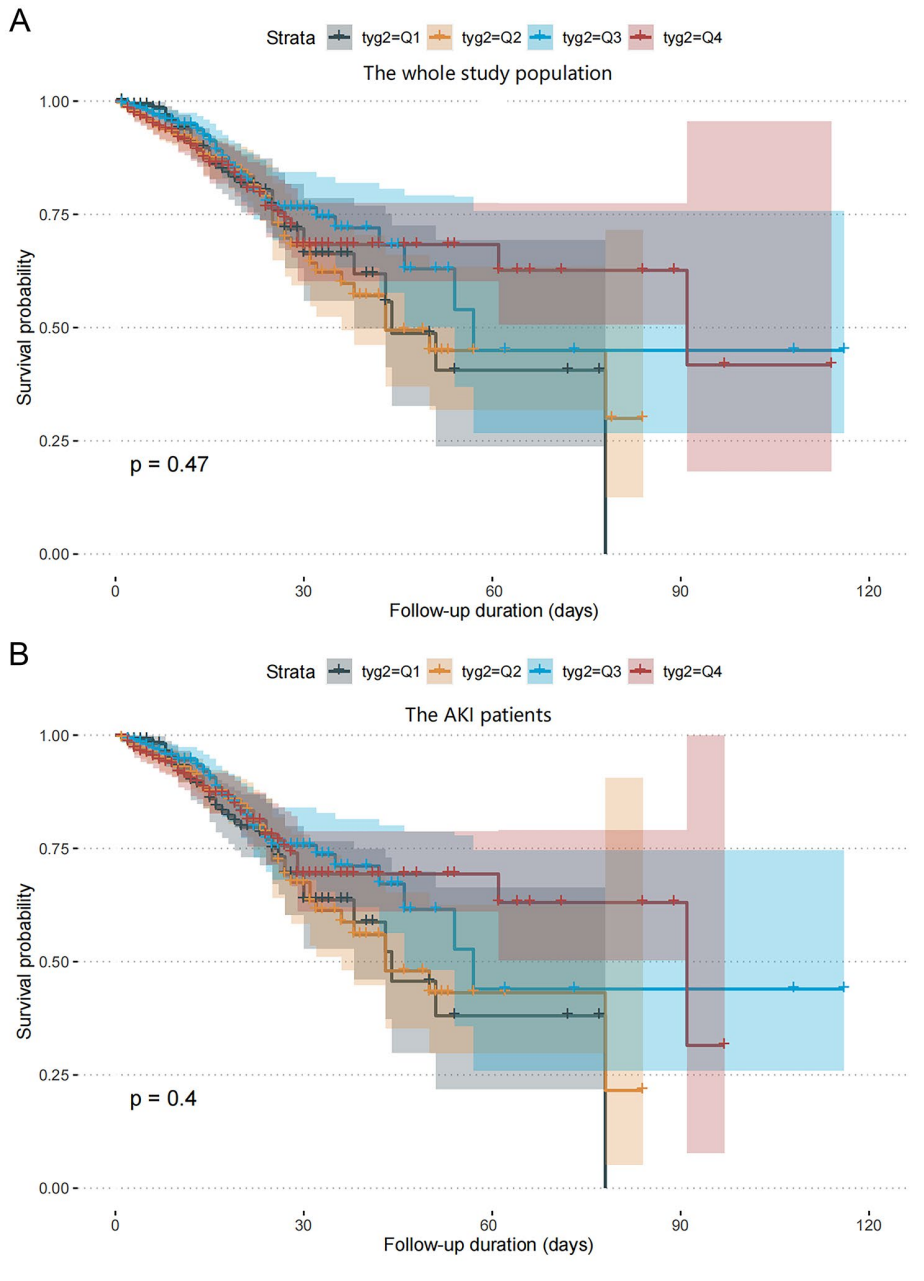
**A**: Kaplan-Meier survival analysis curve for the in-hospital mortality of the whole study population. (TyG index quartile Q1: 3.61–4.61; Q2: 4.61–4.82; Q3: 4.82–5.07; Q4: 5.07–7.18); **B**: Kaplan-Meier survival analysis curve for the in-hospital mortality of the AKI patients. (TyG index quartile Q1: 3.61–4.61; Q2: 4.61–4.82; Q3: 4.82–5.07; Q4: 5.07–7.18)

**Fig. 6:**
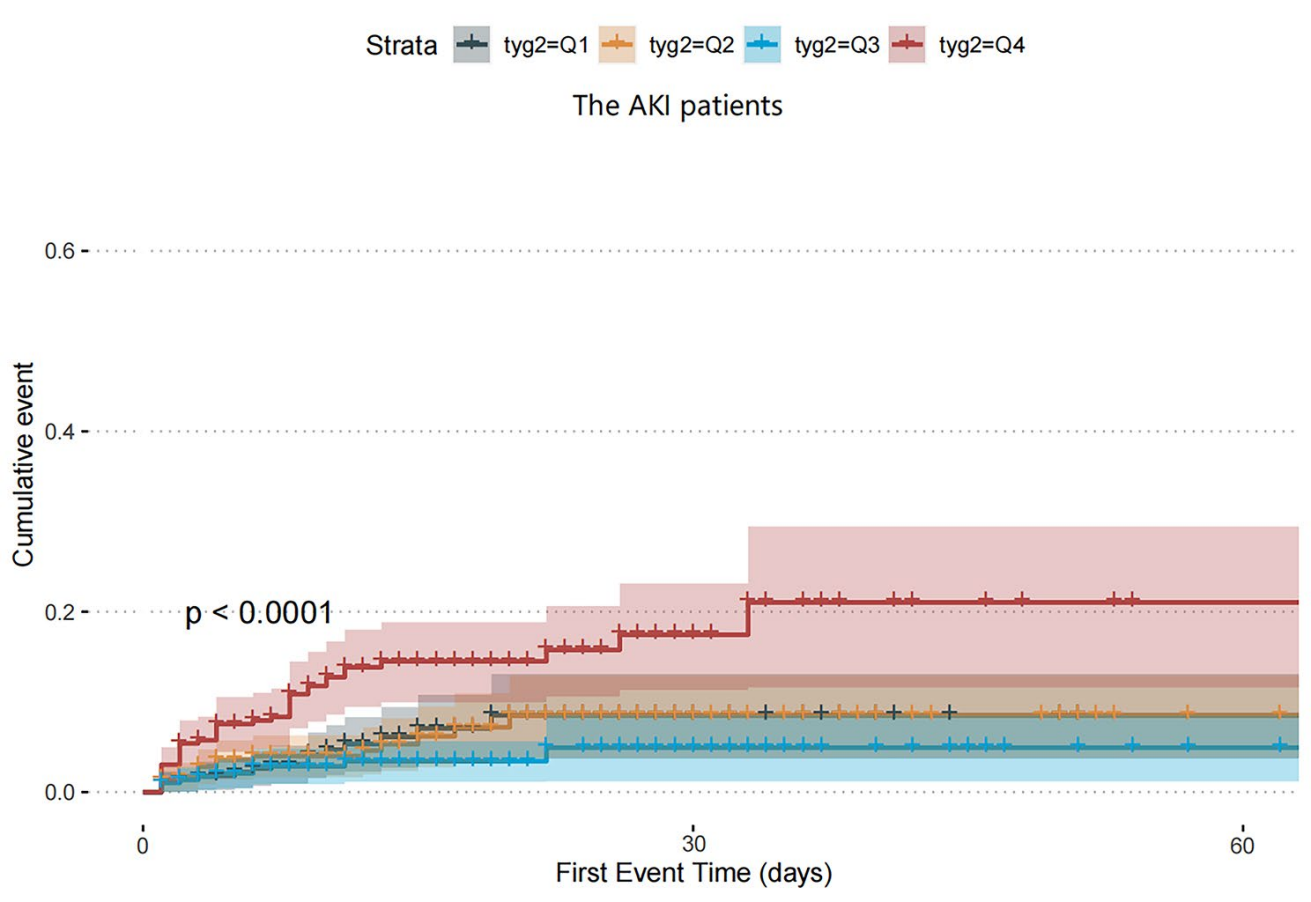
The cumulative event incidence curves for the use of RRT of the AKI patients. (TyG index quartile Q1: 3.61–4.61; Q2: 4.61–4.82; Q3: 4.82–5.07; Q4: 5.07–7.18)

Kaplan-Meier survival analyses were carried out to evaluate the impact of the TyG index on secondary endpoints across the whole study population and the AKI subset. No significant differences were found in the in-hospital mortality between the whole study population (P = 0.47, Fig. 5A) and the AKI subset (P = 0.4, Fig. 5B), based on the TyG index quartiles. However, it was found that AKI patients with the highest quartile of the TyG index faced the highest risk of requiring RRT (P < 0.001, Fig. 6).

## Discussion

To the best of our knowledge, this study represents the first retrospective research investigating the relationship between the TyG index and the risk of AKI in patients with HF in a critical condition. Our findings indicate that patients with HF in a critical condition and elevated TyG index face a heightened vulnerability to AKI. Notably, this association remains statistically significant even after adjusting for potential confounding factors. Additionally, our study underscored a significant association between the TyG index and the progression of AKI to the use of RRT in critically ill patients with HF. Importantly, this research introduces a straightforward methodology for assessing IR to optimize the stratification of AKI risk in patients with HF in a critical condition.

### IR, TyG index, cardiovascular disease (CVD) and kidney disease risk

In spite of the use of treatment strategies and interventions based on clinical practice guidelines, HF continues to be a prevalent and severe condition linked to considerable morbidity and mortality, thus placing an increasing public health burden worldwide [20]. AKI occurs in approximately 47% of patients with HF, especially those critically ill in ICU, and frequently indicates higher short- and long-term mortality [21, 22]. Therefore, there is an urgent need to explore new biomarkers to identify HF patients at a high risk of developing AKI in the ICU to improve their prognosis.

Evidence has revealed the widespread presence of IR in patients with HF or renal impairment, and its occurrence usually precedes the development of HF or renal dysfunction. IR is not only a risk factor for the deterioration of cardiac and renal function deterioration but also influences the incidence of adverse outcomes [23–25].

In previous research, the homeostasis model assessment (HOMA) has been employed as a relatively simple and reliable means of evaluating IR [26]. However, the use of HOMA is associated with high costs, time consumption, and invasiveness, making it less convenient for routine clinical measurements. Consequently, the TyG index was introduced by Unger G et al. in 2013 as a valid, cost-effective, and reproducible indicator of IR [27]. Furthermore, numerous studies have demonstrated the superior performance of the TyG index compared to HOMA-IR [28, 29].

In recent years, a wealth of clinical studies has emerged, highlighting the association between the TyG index and the morbidity and mortality of cardiovascular or kidney diseases across various populations. In the context of cardiovascular diseases, Huang et al. conducted a study revealing the association of a higher TyG index with a higher risk of incident HF and impaired left ventricular (LV) function in asymptomatic individuals without a history of HF and coronary heart disease [30]. Similarly, Park et al. pointed out that the TyG index served as an independent indicator for the presence of coronary heart disease (CHD), particularly mixed coronary artery plaques or non-calcified [31]. Another cohort study conducted by Liu et al. showed that a TyG index exceeding 9.20 was significantly linked to an increased susceptibility to AF in Americans without known cardiovascular diseases [32].

In terms of kidney diseases, Lei et al. pointed out that the TyG index was positively and independently associated with the progression of renal dysfunction in elderly individuals (aged ≥ 65 years) [33]. Additionally, Fritz et al. conducted a large-scale observational study, reporting that increased TyG index mediated the connection between BMI with end-stage renal disease in middle-aged adults [34]. Moreover, many studies have proposed that the TyG index can be a predictive tool for unfavorable prognoses among patients with cardiovascular or kidney diseases. A study by Sun et al., involving 9,254 participants, revealed that the TyG index correlated with overall mortality and cause-specific mortality (malignant neoplasms and CVD) among the middle-aged and elderly population of the United States [35]. Furthermore, a recent study has indicated that a higher TyG index was a predictor for in-hospital and one-year mortality among ICU patients with CKD and CAD [36]. These pieces of evidence collectively emphasize the effectiveness of the TyG index as a dependable and valid marker of IR for risk stratification of AKI among patients with HF in a critical condition.

### Potential mechanisms behind the association of IR and TyG index with AKI in patients with HF experiencing severe illness

Several observational studies have provided evidence suggesting the TyG index can be a predictive factor for the decline in renal function among contrast-induced AKI, diabetic or hypertensive patients, but limited data is available specifically for critically ill patients with HF [15, 37, 38]. Our study presents novel findings demonstrating the strong independent predictive capability of the TyG index for the incidence of AKI in ICU patients with HF. The underlying mechanisms that IR prompts the pathological reaction between heart and kidney may involve the following factors. Firstly, IR is associated with increased glomerular hydrostatic pressure and urinary albumin excretion, which was proved to result in the incidence of early glomerular hyperfifiltration and contribute to late glomerular damage in the early stages of diabetic nephropathy [39]. These hemodynamic burdens and IR may gradually cause injury to the glomeruli and the vasculature supplying them, which are crucial components of renal filtration [40, 41]. Secondly, IR is responsible for the improper activation of sympathetic nervous system and renin-angiotensin-aldosterone system, causing elevated levels of angiotensin II [42–45]. The renal injury induced by angiotensin II is known to be caused by elevated systemic pressure and intrarenal vasoconstriction, resulting in reduced perfusion of the renal tissue [46, 47]. Moreover, excessive activation of sympathetic nervous system can increase cardiac workload and contribute to vascular and renal dysfunction [48]. Thirdly, IR contributes to activating abnormal molecular pathways, such as chronic inflammation, oxidative stress, mitochondrial dysfunction, endoplasmic reticulum, advanced glycation end-products (AGEs), and imbalance of regulation mechanisms, which has been turned out to be harmful to both heart and kidney. [49–51]. Research has established that IR related to oxidative stress can trigger glomerular endothelial cell injury, mesangial cell proliferation, and thickening of basement membranes. These processes collectively contribute to glomerular sclerosis and renal tubular interstitial injury, ultimately culminating in renal insufficiency [52]. In addition, Nakagawa et al. reported that IR resulted in a reduction of nitric oxide synthesis from endothelial cells in the glomeruli, thus promoting the expression of renal vascular endothelial growth factor and increasing marked macrophage infiltration in an animal model [53]. Fourthly, IR can induce changes in substrate metabolism and inefficient energy utilization, thereby hampering the normal myocardial response to injury [54]. Notably, the metabolic efficiency of the myocardium is further dampened in ADHF patients due to the downregulation of genes regulating the beta-oxidation of fatty acids [55]. Moreover, the insulin receptor on renal tubular cells and podocytes plays a pivotal role in insulin signaling, influencing renal hemodynamics, podocyte viability, and tubular function. Defective insulin receptor signaling resulting from IR can induce a pathological condition similar to diabetic nephropathy, even in the absence of high blood glucose levels [56]. Additionally, IR can contribute to heightened sodium reabsorption and an elevated glomerular filtration rate, which could increase cardiac afterload and eventually lead to kidney damage [57]. All these pathophysiological changes collectively contribute to the development of AKI in critically ill patients with HF. Furthermore, HF and AKI mutually contribute to the development of IR, thereby exacerbating the deterioration of cardiac and renal function through a vicious cycle [58–60].

To optimize the risk stratification for AKI in patients with HF in a critical condition, it is imperative to routinely assess the TyG index in this population. This practice will enable early intervention and improve prognosis.

### Study limitations

Several limitations were encountered in this study. First, it should be noted that the study design was retrospective and observational, thereby precluding the establishment of definitive causal relationships. Second, as a single-center study with a limited sample size, despite the use of multivariate adjustment and subgroup analyses, potential data bias might persist due to residual confounding factors. Third, Factors such as the severity of HF and CKD, the etiology of HF, the progression of AKI severity to AKD, CKD, ESRD, baseline characteristics and diagnoses at admission, and the socioeconomic status of study participants were not considered due to constraints inherent in the MIMIC-IV database, which may contribute to potential bias in the study’s outcomes. Fourth, our investigation solely focused on evaluating the prognostic value of the baseline TyG index for AKI in critically ill patients with HF, disregarding any changes in the TyG index. Finally, to validate our findings, prospective cohort studies are necessary.

## Conclusion

In conclusion, this study expands the applicability of the TyG index to the context of ICU patients with HF and establishes that an increased TyG index serves as a predictor and risk stratification tool for AKI in critically ill patients with HF.

## Data Availability

The data that support the findings of this study are available from the corresponding author upon reasonable request.
